# Pilot study of a repeated random sampling method for surveys focusing on date-specific differences in alcohol consumption among university students

**DOI:** 10.1186/s40814-019-0411-z

**Published:** 2019-02-18

**Authors:** John A. Cunningham, Amber Anthenien, Clayton Neighbors

**Affiliations:** 1 Centre for Addiction and Mental Health, 33 Russell St, Toronto, Ontario M5S 2S1 Canada; 2 University of Toronto, Toronto, Canada; 3 University of Houston, Houston, USA

**Keywords:** Student drinking, Epidemiology, Methods

## Abstract

**Background:**

This paper proposes and pilots a repeated random sampling method to promote the likelihood of collecting drinking data equally representative of the behavior of university students at all times through the academic year.

**Methods:**

From October, 2016, to May, 2017, random samples of 1350 students were selected from the 39,155 undergraduate students enrolled in the fall semester at University of Houston. These students were sent an email inviting them to complete an online survey (entered into a weekly draw for a $50 gift certificate if responded).

**Results:**

The response rate was low (6%). Among participants who reported drinking in the last week, there was a variation as expected in the amount of drinking observed depending on the time of year (e.g., during exams).

**Conclusions:**

While the sampling methods show promise, procedures would need to be implemented to substantially increase response rates before the proposed methods could be seen as an advantage over existing survey sampling procedures.

**Electronic supplementary material:**

The online version of this article (10.1186/s40814-019-0411-z) contains supplementary material, which is available to authorized users.

## Background

Epidemiological surveys containing measures of alcohol use [1, 2], in both general population and university student samples, have largely focused on collecting estimates of levels of alcohol consumption as well as their associated consequences [3–5]. Such epidemiological surveys have also been used to describe weekday versus weekend variations in consumption [6, 7]. While some surveys employ sampling methodology that allow for the assessment of variations in consumption at different times during the year (e.g., the CAMH Monitor) [8], the majority of surveys have not focused on time-specific issues relevant to alcohol consumption, such as season, or specific calendar-related heavy drinking events. When epidemiological survey data has been used to track calendar-specific variations in drinking (e.g., Christmas or New Year) [9], the results, while indicative of variations in consumption, suffer from the limitation that the survey sampling methodology does not allow for confidence regarding whether the drinking data produced is equally representative on each calendar date. This is because the survey sampling frame was generated at one (or, at most, several time-points) and then attempts were made to contact participants over an extended period of time. The further away in time from when the sample frame was generated, the greater the likelihood that the participant was hard to reach (displaying systematic differences in demographic characteristics compared to participants who were contacted after one or two attempts) [10, 11]. However, it is important to note that, as calendar-related heavy drinking is not the purpose of these surveys, the lack of information on this topic in these surveys is not a weakness—just a fact relating to the differing purpose associated with their data collection.

One example of a survey tradition that has paid attention to recent drinking events was developed as part of the recurring surveys conducted in Finland [12]. A component of these surveys was a series of questions that asked detailed questions about the participant’s last drinking events. This focus makes particular sense in the Finnish context, at least that context several decades ago, where drinking in Finnish culture was structured around occasional heavy drinking rather than daily, or almost daily alcohol consumption. Another example of a survey focusing on recent heavy drinking events is the “big night out” research conducted by Dietz and colleagues [13].

In the general population, the exploration of event-specific drinking patterns has largely relied on relating temporal trends in alcohol sales data to alcohol-related hospital admissions, ambulance attendances, or deaths [14, 15]. In addition, studies have been conducted that link survey data to other registers, such as those recording cause of death [16]. In university settings, research has more often employed convenience samples, relating increases in alcohol consumption to specific social events (holidays, birthdays, and sporting events) [17–20] or to follow a convenience sample longitudinally and note the variation in their consumption over time [21–24]. While both of these approaches have their strengths and limitations, there is merit in considering other methods of collecting drinking data that are calendar-specific and which pay attention to the representativeness of the sampling frame across time. This would allow for increased confidence that the detailed information collected through convenience samples is generalizable if the same patterns of results are observed when epidemiological sampling techniques are employed.

### Aims and objectives

This paper proposes and pilots a survey methodology of repeated random sampling. The goal was to target the academic year of the entire undergraduate population of a university. Student drinking was chosen as an example because of the variable nature of their drinking over the academic year [21]. Further, studying drinking patterns during the university period is important because it is a time when drinking patterns are established that can continue through extended periods of the person’s lifespan [25]. Many students drink in an episodic fashion, with heavy drinking incidents occurring around specific events in the university academic year. In addition, some of the stressors of student life (e.g., exams, assignments) also occur in a systematic pattern across the school year. This means that there may be times of the academic year where similar quantities of alcohol consumption could cause greater or lesser harm as well as variable levels of mental distress. Accurate estimates of student drinking over the academic year could prove particularly useful for targeted interventions, such as event-specific normative feedback [26]. The proposed survey sampling method should allow for the creation of a day-by-day summary, tracking the incidence and co-occurrence of drinking, mental distress, and negative consequences.

## Methods

This pilot study was conducted for the period from mid-October to the end of April. We accessed the email list of the 39,155 undergraduate students registered in the fall of 2016 at the University of Houston. From this population, random samples (without replacement) of 1305 potential participants were selected weekly and were sent a link asking them to participate in a student life survey (email invitation sent on Fridays; all undergraduate students were sent an email invitation at some point during the pilot study period; the randomization for this pilot employed the randomization feature provided in the software program housing the email list; see Additional file 1: Appendix 1 for a copy of the email invitation). The email contained a link to a brief description of the study, an electronic consent form, and the survey itself. Potential participants were informed that those completing the survey each week would be entered into a draw for a $50 Amazon.com coupon.

### Why not randomize with replacement?

One alternate sampling design would be to randomize with replacement (i.e., the emails that are selected each week are “replaced” back onto the full list of emails so that they have an equal chance of being selected again when the list of emails is selected the next week and so on). While this is a “purer” form of randomization, it was judged to be more feasible to employ random sampling without replacement (i.e., the emails, once selected, are not added back onto the list of emails) in order to ensure that all undergraduate students would have the opportunity to participate and to reduce response burden (and the concomitant likelihood of reduced response rates) resulting from some students being asked to fill out a survey on multiple occasions throughout the academic year.

### Survey content

The primary content of the online survey was:

1. Demographics characteristics: age, sex, year of undergraduate study, live on or off campus, and ethnic background using NIH categories.
2. Measurement of health-related quality of life (HRQOL) using the EUROHIS-QoL8. [27].
3. Smoking variables: whether smoked cigarettes daily, occasionally, or not at all in the past 12 months; number of cigarettes usually smoked each day; and time upon waking to smoking first cigarette.
4. Drinking variables: frequency of alcohol consumption in the last year (including no alcohol use option), number of drinks on each day of the previous week, the AUDIT-C three-item alcohol consumption scale that estimates severity of alcohol consumption [28, 29].
5. Those who consumed alcohol in the last week were asked about the experience of any alcohol-related problems using the 18-item version of the Rutgers Alcohol Problem Index (RAPI) [30].
6. Measurement of psychological distress using the Kessler 10 (K10) [31].

## Results

Of the 39,155 emails sent, only 2432 (6%) of students replied to the email, consented to the study, and provided responses on the survey. While the number of email invitations sent each week was the same (*n* = 1305), the number of participants responding each week varied, ranging from 18 to 133. Further, while all email invitations were sent out on the Friday of each week, the number of participants who replied on each day of the week clustered in a surprising fashion (particularly as there was no mention of a time limit within which participants needed to respond that had been provided with the email invitation). The largest proportion of participants replied on the Friday (28.7%), 23.8% replied on Tuesday, and 22.9% replied on Thursday.

Table 1 provides a summary of the demographic characteristics of the participants who responded and of the entire 39,155 students registered for the 2016 fall semester. It appeared that, while the average age of respondents who responded to the survey was roughly similar to the student undergraduate population, males may have been less likely to respond as well as those in the senior academic year.

**Table 1:** Demographic characteristics of survey sample and of those sent the survey invitation email

	Completed survey (*n* = 2432)	Sent survey invitation email (*n* = 39,155)
Mean (SD) Age	22.1 (5.0)	23.1 (5.1)
% Male	40.3	50.8
% White	32.1	25.8
Year of undergraduate studies		
% Freshman	19.2	15.2
% Sophmore	21.7	22.8
% Junior	29.9	26.1
% Senior	26.7	32.0
% Other/Missing	2.5	4.0

**Fig. 1:**
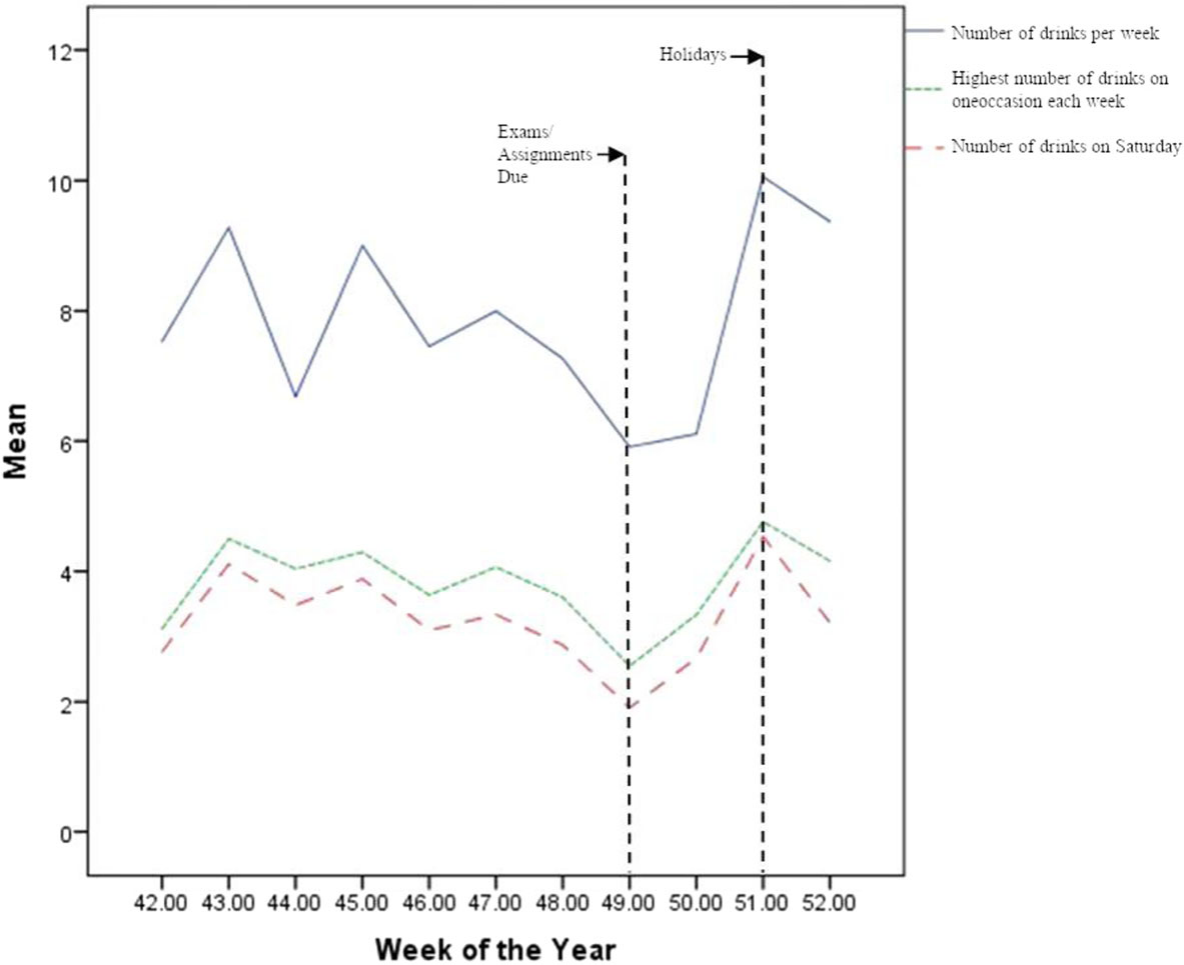
Pattern of weekly alcohol consumption reported from October 14 to December 31, 2016

A total of 1461 participants reported drinking alcohol in the last year and 685 reported drinking something on at least one of the days in the last week. Figure 1 displays the weekly variation in average weekly consumption, highest number of drinks consumed on one occasion during the last week, and the number of drinks consumed on the Saturday of each week (weeks 42–52 presented, with week 42 starting on Friday, October 14th, 2016). As can be observed, there was some weekly variation in alcohol consumption, with the lowest quantities reported being in weeks 49 and 50 (the point in the year where major assignments were due and exams were conducted). The largest quantities reported during the period presented on Fig. 1 appeared to be participants who replied to their surveys in week 51, which started on Friday, December 16th. See Additional file 2: Appendix 2 for a copy of drinking patterns across the full period of data collection.

## Discussion

The goal of this project was to pilot a sampling method that promoted the likelihood of the collection of survey data that was equally representative of the behavior of students at all times through the academic year. In the worst case, one might argue that this was a failed pilot project because the response rate was so low (6%) that discussions of representativeness are not meaningful [32]. The pilot could also be seen as having some successful elements because it demonstrated that the proposed technique was feasible to implement. In addition, even with the low response rate, the expected variations in alcohol consumption could be observed (e.g., reduced alcohol consumption around the time of exams and final assignments).

There are a number of limitations associated with the proposed sampling method and this pilot trial. As with other population surveys, there is the likelihood of a poor response rate. This could be offset to a certain extent by employing techniques to promote participation in online surveys of this type in undergraduate populations [33]. These methods include advertising widely in the study population that the survey was occurring, sending out a paper letter to participants prior to the email invitation to alert them that they will be receiving an email invitation, more aggressive follow-up of nonresponders, and reimbursing participants for completing the survey. It would also have been valuable to collect some qualitative data from students who did not respond to get an idea as to why they were choosing not to participate. Also of concern is the possibility that response rates would vary across the academic year, with low response rates during exam times and holidays. This would lead to challenges with interpreting differences in drinking patterns observed across the calendar year. Further, for the current pilot trial, we did not attempt to collect data during the very start of the academic year. The orientation period both would be important to monitor because of the heavy use of alcohol during this period [25], but also might be a more challenging period to conduct the survey (e.g., getting access to a finalized student email list). Finally, while there were multiple random samples generated throughout the school year, there is some potential for a differential level of recall bias on some days of the week versus others (e.g., recall of the previous Monday compared to the previous Sunday). This is because the time when a link to the survey was sent to participants varied in a systematic fashion across the week (sent on Fridays). Still, the recall bias for past week drinking is likely to be considerably lower than assessments that ask students to report drinking over the past month or past 3 months.

### Alternate design

An alternate design would have been to select random samples on every day of the academic year as opposed to just once a week. In this alternate version, the survey would ask about the occurrence of the variables of interest over each day of the previous week up to and including the previous day. The analysis process would, as its first step, compile the responses of participants by matching up participants’ responses specific to each calendar date that they are asked about. Thus, data collected for each specific date would consist of up to *X* participants discussing their activities from the previous day (number of participants depends on size of the university student population), an additional potential *X* participants discussing their activities from the day 2 days earlier, etc. One complexity with implementing this design, as opposed to the weekly sampling method tested here, is that it would be more important to be sure of the dates for each of the days the participants responded to (i.e., so the data could be compiled for analysis). Further, there would be challenges to implementation and interpretation if participants did not respond to the survey request on the day it was sent but instead wanted to complete it at a later date.

### Examples of research questions benefitting from data collected in this manner

The specific variables chosen to include in the survey could be modified to optimize the collection of data addressing the particular research question under study. There are a number of research questions that would benefit from access to data collected in this manner. These include the possibility of mapping date-specific drinking and drug use to social events occurring at the university and to control initiatives implemented by university staff. It would also be possible to match drinking patterns to disturbances recorded by campus security or local police. Data collected using a repeated random sampling method could also be employed to track patterns of alcohol use and level of mental distress over the academic year (note: while mental health distress was assessed in this pilot survey, these analyses were not conducted as the response rate was too low for any results to be meaningful). Similar analyses could be conducted to track the co-occurrence of drinking with other health behaviors (e.g., smoking) or with health-related quality of life. This aggregated, date-specific data would complement longitudinal research on this topic which employed convenience samples of participants [21]. Further, means of multiple random samples of students reports of past week drinking represents a sampling distribution. In comparison to single annual or biannual assessments of campus drinking (assuming similar participation rates), estimates derived from a multiple samples provides a more complete and accurate representation of typical drinking on campus. This approach also allows for the direct calculation of standard errors whereas single assessments only provide estimated standard errors. Finally, accurate data of patterns of alcohol consumption during date-specific drinking events could be used to generate population norms for personalized feedback interventions targeting people who drink in a hazardous fashion.

## Conclusion

While the proposed repeated randomization method does not address all limitations, it could provide a survey sample that is more representative of drinking on each calendar day compared to other population sampling methods. The method could also work in a general population setting if the researcher had good access to contact data for residents of the country that would allow for a similar repeated sampling strategy (e.g., voter registration files) and a method for delivering the surveys on a daily basis.

## Additional files

Additional file 1: **Appendix 1.** Text for initial email invitation. (DOCX 2265 kb)

Additional file 2: **Appendix 2.** Drinking data from entire period of data collection . (DOCX 2065 kb)

## Abbreviations

AUDIT-C: Alcohol Use Disorders Identification Test Consumption scale
HRQOL: Health-related quality of life
K10: Kessler 10
RAPI: Rutgers Alcohol Problem Index
