# Supplementary material for: Pilot study of a repeated random sampling method for surveys focusing on date-specific differences in alcohol consumption among university students
Source: Pilot Feasibility Stud. 2019 Feb 18;5:26. doi: 10.1186/s40814-019-0411-z (PMC6378749; doi:10.1186/s40814-019-0411-z)
Supplement: Supplementary file 1 — Appendix 1. Text for initial email invitation. (DOCX 2265 kb) [file 40814_2019_411_MOESM1_ESM.docx]

**Appendix 1: Text for initial email invitation**

***Subject line:* Help us out with a brief survey on student life**

Hello, we are conducting a brief survey on student life. We want to know how you are feeling, and how common drinking and smoking cigarette is among University of Houston students. We would like you to participate even if you don’t drink or smoke because we are trying to get a picture of what is going on for the whole student population. At some point during the year, every student will receive one of these email requests. Your email was selected by chance this week.

Please click this link to fill out the brief survey: *Insert link here*

Once you have completed the survey, we will enter you into this week’s weekly draw to win a $50 gift certificate from Amazon.com. Participation in this survey is voluntary but it would be very helpful for us if you filled it out for us.

Thanks,

The Student Live Research Team

A joint project of researchers from the University of Houston and the University of Toronto
