# Supplementary material for: Pilot study of a repeated random sampling method for surveys focusing on date-specific differences in alcohol consumption among university students
Source: Pilot Feasibility Stud. 2019 Feb 18;5:26. doi: 10.1186/s40814-019-0411-z (PMC6378749; doi:10.1186/s40814-019-0411-z)
Supplement: Supplementary file 2 — Appendix 2. Drinking data from entire period of data collection . (DOCX 2065 kb) [file 40814_2019_411_MOESM2_ESM.docx]

Appendix 2: Drinking data from entire period of data collection


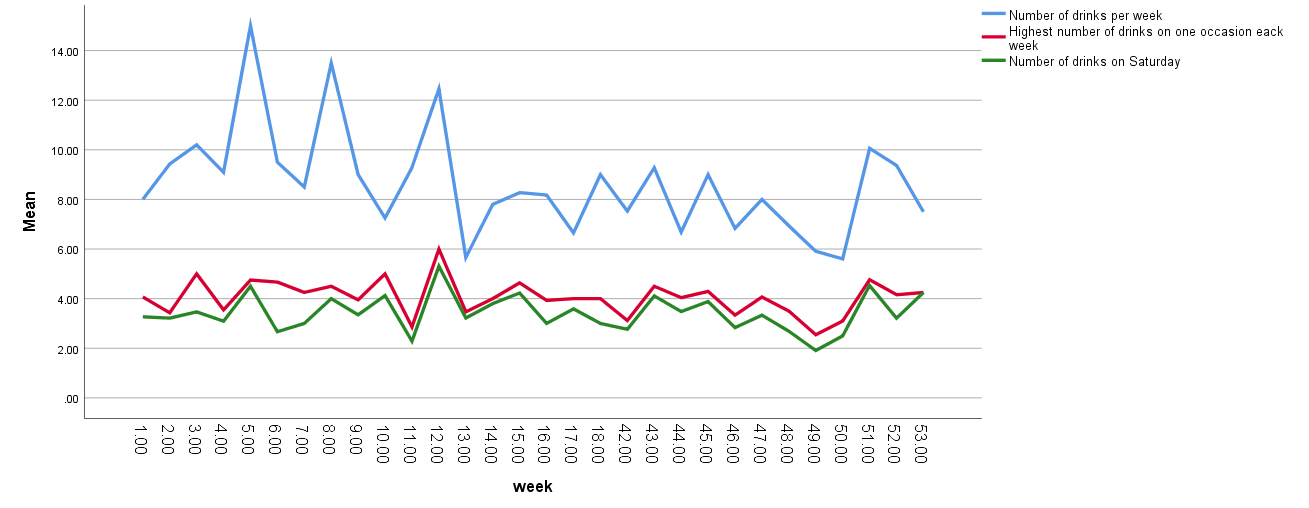


Note: Weeks 1-18 for the first weeks in 2017. Weeks 42 onwards are the last weeks in 2016.
